# Involving service users in trials: developing a standard operating procedure

**DOI:** 10.1186/1745-6215-14-219

**Published:** 2013-07-17

**Authors:** Bridie Angela Evans, Emma Bedson, Philip Bell, Hayley Hutchings, Lesley Lowes, David Rea, Anne Seagrove, Stefan Siebert, Graham Smith, Helen Snooks, Marie Thomas, Kym Thorne, Ian Russell

**Affiliations:** 1 College of Medicine, Swansea University, Swansea, Wales, UK; 2 Clinical Trials Research Centre, University of Liverpool, Liverpool, UK; 3 Service user, Anglesey, Wales, UK; 4 School of Nursing and Midwifery Studies, Cardiff University, Cardiff, Wales, UK; 5 College of Human and Health Sciences, Swansea University, Swansea, Wales, UK; 6 College of Medical, Veterinary and Life Sciences, University of Glasgow, Glasgow, UK; 7 Faculty of Education and Social Services, University of Wales, Newport, Wales, UK

**Keywords:** Consumer participation, Consumer involvement, Clinical trials, Service user involvement

## Abstract

**Background:**

Many funding bodies require researchers to actively involve service users in research to improve relevance, accountability and quality. Current guidance to researchers mainly discusses general principles. Formal guidance about how to involve service users operationally in the conduct of trials is lacking. We aimed to develop a standard operating procedure (SOP) to support researchers to involve service users in trials and rigorous studies.

**Methods:**

Researchers with experience of involving service users and service users who were contributing to trials collaborated with the West Wales Organisation for Rigorous Trials in Health, a registered clinical trials unit, to develop the SOP. Drafts were prepared in a Task and Finish Group, reviewed by all co-authors and amendments made.

**Results:**

We articulated core principles, which defined equality of service users with all other research team members and collaborative processes underpinning the SOP, plus guidance on how to achieve these. We developed a framework for involving service users in research that defined minimum levels of collaboration plus additional consultation and decision-making opportunities. We recommended service users be involved throughout the life of a trial, including planning and development, data collection, analysis and dissemination, and listed tasks for collaboration. We listed people responsible for involving service users in studies and promoting an inclusive culture. We advocate actively involving service users as early as possible in the research process, with a minimum of two on all formal trial groups and committees. We propose that researchers protect at least 1% of their total research budget as a minimum resource to involve service users and allow enough time to facilitate active involvement.

**Conclusions:**

This SOP provides guidance to researchers to involve service users successfully in developing and conducting clinical trials and creating a culture of actively involving service users in research at all stages. The UK Clinical Research Collaboration should encourage clinical trials units actively to involve service users and research funders should provide sufficient funds and time for this in research grants.

## Background

Actively involving service users in health and social care research is encouraged as a way to improve research quality, relevance and accountability [1-3]. The Department of Health and devolved administrations, notably the Welsh Government, recommend it as good practice. Many health research funding bodies now require information on their application forms about the extent and methods of involving service users in designing and undertaking research [4-6] (http://www.hta.ac.uk/public/getinvolved/conductingresearch.shtml or http://www.sdo.nihr.ac.uk/getinvolved.html). The Central Commissioning Facility of the National Institute for Health Research states on its website that “applications that are technically excellent but have little patient or public involvement may be asked to address this before an offer of funding is made” (http://www.nihr-ccf.org.uk/site/consumerinvolvement/infoforresearchers/default.cfm).

Service users bring understanding and experience of conditions and interventions to the process of designing and conducting trials [5,7]. We use the term ‘service user’ to refer to patients, carers and people eligible for a service, whether or not identified or diagnosed, and anyone else considered relevant to the study inclusion criteria [8].

Reports of impacts include influencing the development of research studies (e.g. shaping research questions); the choice of methods for trials (e.g. by helping to select the content of control arms); making the research more ethical and encouraging recruitment (e.g. by helping explain different concepts in patient information to enable informed consent); widening dissemination (e.g. by identifying non-academic routes and the best format for communicating findings) [3,9-17]. Involving service users can increase public understanding of the purpose and process of science and can help make researchers more accountable to the people who may use services and contribute through taxes and donations [3].

Service users contribute to both the content and methods of research, including developing, reviewing and commissioning research proposals and undertaking research using a wide range of methods [13,18-26]. Health Technology Assessment International (HTAi) has a Citizen and Patient Involvement group that aims to embed public and patient involvement across its membership of 59 countries (see http://www.htai.org/index.php?id=545) and public involvement in international Health Technology Assessment activities is growing [27]. Work is underway to enhance the reporting of public and patient involvement in health research through GRIPP 2 with EQUATOR to strengthen the evidence base about service user involvement in research (see http://www.equator-network.org/resource-centre/library-of-health-research-reporting/reporting-guidelines/other-reporting-guidelines/). Although researchers are increasingly involving service users in planning and undertaking research, there is limited engagement with clinical trials [3,28].

Guidance on how to involve service users in research mainly discusses general principles (e.g. [29]). Recent guidance by INVOLVE outlines benefits and challenges of engaging service users in trials [30]. However, formal guidance about how to involve service users operationally in the conduct of trials is lacking although guidance for service users has recently been prepared (see http://www.trialsjournal.com/imedia/1292821605618248/supp1.doc). Recent evidence reports the importance of context and process on involvement, highlighting the need for researchers to understand their role in facilitating service user involvement and follow best practice [13,31]. Researchers can be unsure how to involve people in research and need access to information and support in order to understand the potential opportunities and how to manage the involvement process with clarity and consensus [32]. There is also a need to tackle misunderstood anxieties around who to involve, to explain the contribution of service users’ perspectives, so that researchers are not misled by anxieties about gaining representation, the so-called ‘red herring’ [33], in place of experience [32,34,35].

The West Wales Organisation for Rigorous Trials in Health (WWORTH) is a clinical trials unit based in Swansea University; it has recently received provisional UK Clinical Research Collaboration (UKCRC) registration following, among other things, the development of a suite of Standard Operating Procedures (SOPs). Although UKCRC did not require an SOP on involving service users before awarding registration, we judged that such an SOP would enhance researchers’ ability to involve service users throughout development and implementation of trials and other rigorous studies. We believed it would provide legitimacy for involving service users in trials and provide a starting point on which researchers could build. We considered it would also benefit the conduct and general quality of such studies. Other WWORTH SOPs, now numbering more than 30, cover project management, qualitative methods, the use of routine data and more familiar topics like randomisation, data management and statistical analysis.

Academics and service users together developed the SOP for involving service users to provide guidance to researchers. The authors have considered how to involve service users actively in trials and rigorous mixed-methods studies, and in developing research ideas through trial development groups. Using our experience, we aimed to ensure that the involvement of service users was in line with best practice and maximised opportunities to improve the design, conduct, analysis and dissemination of research [36,37]. We adhered to elements of the GRIPP checklist where relevant to reporting guidance [38].

## Methods

Researchers at Swansea University with experience in actively involving service users joined one of three Task and Finish (TAF) groups responsible for developing WWORTH’s initial portfolio of SOPs. Conscious that ‘patient involvement’ can be passive, even tokenistic, we chose to entitle our SOP ‘Involving service users’. Thereafter the SOP followed a standard process: selected TAF members drafted the SOP in collaboration and other group members reviewed and amended successive drafts. Three service users with experience of contributing to trials and three academics with experience of actively involving service users in research, including trials, then reviewed the penultimate draft, leading to further amendments. Two members of the WWORTH Development Group, which coordinated the registration application to UKCRC, independently reviewed the final draft. The Development Group approved the SOP for use following discussion at a regular meeting. The SOP development process is shown in Table 1.

**Table 1:** Process of developing the SOP

**Key steps**	**Who was involved**
Step 1: drafting SOP	Researchers with experience of involving service users in research + Clinical Trial Unit staff
Step 2: reviewing draft SOP	Service users + external academics with experience of involving service users in research
Step 3: approving SOP	Service users + researchers with experience of involving service users in research + Clinical Trial Unit staff

We based this SOP on research evidence, good practice and experience of actively involving service users in research, including randomised trials [9,20,39-42]. We aimed to define a process that addressed service users’ needs [43-45] and also ensured their partnership within the research process from the development of a proposal and involvement as co-applicants, through undertaking the study, to disseminating results in a range of formats.

For us, the term ‘involvement’ epitomises the process of actively involving service users within the research process and taking full consideration of their contributions at all stages of trial development and implementation, interpretation of results and dissemination. This is in line with INVOLVE’s definition of public involvement in research as research being carried out ‘with’ or ‘by’ members of the public rather than ‘to’, ‘about’ or ‘for’ them [36]. Involvement is different from participation, where people take part in a research study, and engagement, where information and knowledge about research are provided and disseminated [36]. We expected that service users would collaborate and share decisions with other partners. Collaboration is defined as an approach characterised by partnership between researchers and members of the public and shared decision-making. It is the second level of INVOLVE’s involvement continuum (consultation – collaboration – user controlled). We also described opportunities for involvement by consultation to inform a process of collaborative or shared decision-making since involvement in a project can vary between approaches [32,36,37]. The SOP therefore assumes service users are involved in trials through collaboration and consultation.

## Results

We articulated the key principles that underpinned the SOP:

▪ Service users’ knowledge is a valuable contribution to the formulation of evidence-based medicine and should contribute to trials whenever possible

▪ Service users should contribute at all stages of a trial, from germination of an idea to dissemination of results

▪ Service users should have equal status with other research team members for the expertise they bring; all team members’ views should receive equal consideration

▪ Like all team members, service users should receive the information and practical assistance necessary to undertake their role

We recommend that trial development groups formalise these principles in their Terms of Reference when starting to involve service users within a trial team to articulate shared and differing motivations and vision. The SOP, which can be viewed at http://www.trustresearch.org.uk/en/publications.htm, also provides guidance on how to achieve these key principles, divided into three sections. Each element of the SOP links back to source evidence, allowing the reader to gauge provenance and seek further information.

### SOP guidance

#### Section 1: planning the process of involving service users

We advise that researchers understand the benefits of involving service users in trials and how to do so. The reasons for involving service users and the research aims and objectives should inform decisions on who to involve and how to identify them through patient groups or charities, either focusing on the condition in question or generic Public and Patient Involvement groups and voluntary associations. We highlight practical ways of enabling involvement like: reimbursement of costs; payment of honoraria; selection of suitable venues; appropriate meeting arrangements and information formats; training and support for service users; and provision of sufficient time, expertise and financial and other resources to enable service users to contribute successfully to trials. We propose that researchers protect a minimum of 1% of their total research budget to support service users and build sufficient additional time into proposed timescales to allow for careful recruitment and active involvement of service users. This equates to £10,000 of a £1 million trial or £20,000 of a £2 million trialThis should be allocated for expenses to include honorariums to take part in meetings, out of pocket expenses, the provision of training, support and other costs to facilitate service users’ full involvement over the life of a trial.

#### Section 2: implications for trial management of involving service users

**Figure 1:**
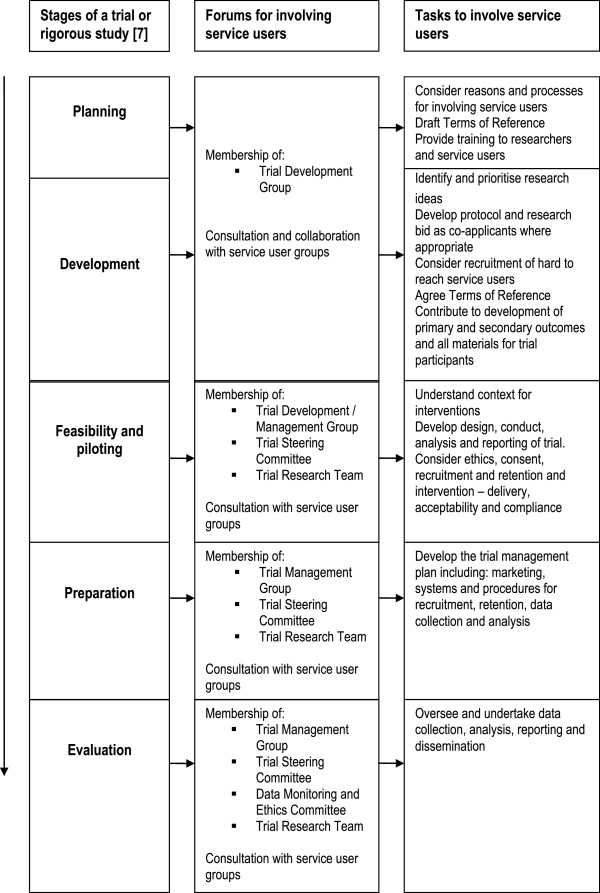
Flow diagram for including service users in trials and rigorous studies.

We list the people responsible for involving service users within trials and promoting a collaborative culture based on mutual respect, courtesy and equal opportunity. We ask the Chief Investigator to take general responsibility for implementing the SOP across the trial, and the Trial Manager or Trial Coordinator to take specific responsibility for supporting and liaising with the recruited service users. The SOP recognises other models exist and could be considered to support service users in trials, such as costing (for example, 5%WTE) and naming a ‘service user involvement lead’. The SOP advocates that research staff should have an understanding of how to support service users and provide training in this topic if necessary. This training should complement training to enable service users to contribute to research. Training for all trial members should be itemised within the trial budget. Additionally, Clinical Trial Units (CTU) should ensure staff are trained in service user involvement to undertake their CTU roles. We also advocate engaging service users as early as possible in the research process and including a minimum of two people on all formal trial groups and committees, in line with recognised good practice [2,6,29]. We recommend recruiting a third service user who also receives training and regular briefings in order to remain engaged and well informed and can cover for inevitable absences. In this way, the research team will maximise the likelihood that two service users can be effectively involved throughout the trial. Table 2 lists the roles and status of service users. We require service users to contribute to decisions about all aspects of planning, designing, conducting and reporting a trial, such as selecting outcomes, designing data collection tools, reviewing ethical standards, undertaking analysis and disseminating beyond academic audiences, as detailed in Figure 1. We ask that trial meetings and documents avoid acronyms and clinical terms where possible to facilitate equal participation of all and that Service Users be a standing agenda item to ensure that research teams hear and address relevant issues.

**Table 2:** Role and status of service users at each trial management level

**Level of management**	**Service user role**	**Service user involvement**
Trial Steering Committee (TSC)	Contribute to corporate decision-making about design, conduct and reporting	WILL involve two service users
Data Monitoring and Ethics Committee (DMEC)	Contribute to corporate decision-making about safety, benefit and recruitment	SHOULD involve two service users but not mandatory as DMEC reports to TSC
Trial Development Group (TDG)	Contribute to corporate decision-making about all aspects of proposed trial	WILL involve two service users
Trial Management Group (TMG)	Contribute to corporate decision-making about all aspects of funded trial	WILL involve two service users
Trial Research Team (TRT)	Collaborate in generic teamwork across trial	SHOULD involve two service users
Ad hoc operational meetings	Collaborate in task-oriented team work across trial	SHOULD involve two service users when feasible

#### Section 3: processes for involving service users in trials

Figure 1 displays our flowchart for involving service users in research, derived from the Medical Research Council’s framework for developing and evaluating complex interventions in health [7]. At each stage, we define a minimum level of involvement and propose additional opportunities such as specific service user groups, collaborative workshops and open meetings to enable service users to contribute at a level of detail and engagement not always feasible in trial development and management groups. In doing so, all co-authors recognise that different service users can best participate in different ways in order to bring to the research a range of perspectives based on different sources of information and evidence. Hence we seek to provide adequate opportunities for experiences that are relevant to the individual trial to be shared within the research team.

## Discussion

Effectively involving service users benefits research [3]. In practice, however, seeking to involve service users encounters practical issues and unreceptive attitudes [22,32,46]. While SOPs cannot themselves overcome these barriers, or prevent tokenistic ‘involvement’, they can provide an appropriate framework, guide researchers about the processes required for good practice, and help to create a culture that expects to involve service users at all stages of the research process and is conducted to ethical standards [28,47]. Despite the emerging evidence base and strong policy support [48], the legitimacy of involving service users in research is still questioned and it still happens in a minority of research studies [49,50]. Our SOP recognises the moral justification for, and benefits of, including service users. It describes an expected minimum standard and culture to guide researchers to involve service users in trials and encourage them to further involvement. It also makes specific recommendations for providing the financial and time resources necessary to achieve effective involvement and foster an environment to support researchers in understanding its potential and current limitations [10,19,43-45,51]. It delineates the role of service users across all the structures and functions of a trial and thus establishes them as equal and legitimate members of a research team. Implementation of our SOP will enable the guidance to be tested and reviewed.

This SOP proposes that research teams allocate at least 1% of proposed trial financial budgets to the training and support of service users and ensure that they have enough time within their busy schedules to provide effective training and moral support to enable collaboration. Funding bodies should expect proposed budgets and timetables to cover these additional resources, respond sympathetically to research proposals that do so and ensure that research awards allocate sufficient funds and time to underpin the effective involvement of service users.

## Conclusion

Intention to involve service users actively in trials and other rigorous studies in health and social care is an explicit requirement in all major research funding bids and has become an essential feature of good research practice. We offer this SOP to guide researchers in successfully involving service users in developing and conducting clinical trials. We propose that UKCRC should require all clinical trial units seeking registration or re-registration to show how they will involve service users in their work by preparing a specific SOP within their portfolio of SOPs. We also propose that funding bodies should encourage researchers to build sufficient funds and time into research proposals to involve service users effectively. These steps will encourage good practice in involving service users in research and help to improve relevance, accountability and quality of health and social care research.

## Abbreviations

WWORTH: West Wales Organisation For Rigorous Trials In Health; SOP: Standard operating procedure; UKCRC: United Kingdom clinical research collaboration; TAF: Task and finish; TSC: Trial steering committee; DMEC: Data monitoring and ethics committee; TDG: Trial development group; TMG: Trial management group; TRT: Trial research team; CTU: Clinical trials unit.

## Competing interests

The authors declare that they have no competing interests.

## Authors’ contributions

BAE wrote the first draft of the Standard Operating Procedure and was responsible for revisions. HH, IR, AS, SS and MT were members of the authorship team. EB, PB, LL, DR, GS, HS and KT reviewed and commented on the SOP. BAE wrote the first draft of this manuscript and was responsible for the revisions. All authors provided input into drafting the manuscript and read and approved the final version.
